# Differentiating diabetes type in children and adolescents with ketosis or ketoacidosis at onset: a retrospective analysis of clinical and biochemical markers

**DOI:** 10.1186/s12902-025-02077-x

**Published:** 2025-11-12

**Authors:** Hongxia Liu, Yan Wang, Miao Wang, Bo Zhang, Caixia Ma, Lianlian Cui, Qianhan Wang, Zhenfeng Cao, Zhongwen Yang, Changsong Shi

**Affiliations:** Department of Pediatrics, Henan Provincial People’s Hospital, People’s Hospital of Zhengzhou University, People’s Hospital of Henan University, Zhengzhou, Henan 450003 China

**Keywords:** Diabetic ketosis (DK), Diabetic ketoacidosis (DKA), Clinical characteristics, Diabetes typing, Adolescents

## Abstract

**Background:**

Distinguishing diabetes diagnosis is fundamental to ensuring proper management of individuals with diabetes, but has been challenging, especially in newly diagnosed diabetes onset with ketosis or ketoacidosis in pediatrics.

**Methods:**

A retrospective analysis was conducted on medical records from 2017/1/1 to 2020/4/30 in pediatrics with new-onset diabetes accompanied with ketosis or ketoacidosis. Data was collected at diabetes onset and two years after discharge. Persons with diabetes were classified as type 1 or 2 diabetes (T1DM; T2DM) based on the person’s medication and final diagnosis. The best diagnostic cut-off point was determined using receiver operating characteristic curves (ROCs) between T1DM and T2DM.

**Results:**

Among 153 children and adolescent with diabetes, 78 (51.0%) were diagnosed as T1DM and 75 (49.0%) were diagnosed as T2DM after two years of follow-up. There were significant differences in sex, age, family history, BMI, systolic and diastolic blood pressure, lipids, uric acid (UA), C-peptide, combined fatty liver ratio and any islet auto-antibody-positive ratio at the time of onset (*P* < 0.05). Key discriminators identified by ROC analysis included fatty liver, SBP, BMI, and C-peptide levels (fasting, 1-h, and 2-h), with AUCs of 0.79, 0.83, 0.92, 0.94, 0.96, and 0.95 and optimal cut-offs value of 110.5 mmHg, 20.95 kg/m², 0.47 nmol/L (fasting), 0.98 nmol/L (1-h), and 2.03 nmol/L (2-h), respectively.

**Conclusions:**

Overall, the most sensitive and specific clinical and biochemical criteria for the diagnostic classification of newly diagnosed diabetes onset with ketosis or ketoacidosis in pediatrics should consider C-peptide, BMI, SBP and fatty liver at the time of onset, which have effective diagnostic values.

**Clinical trial number:**

Not applicable.

**Supplementary Information:**

The online version contains supplementary material available at 10.1186/s12902-025-02077-x.

## Highlight

Distinguishing diabetes diagnosis is fundamental to ensuring proper management of individuals with diabetes, but has been challenging, especially in newly diagnosed diabetes onset with ketosis or ketoacidosis.

The current study retrospectively collected the clinical data from 153 newly diagnosed diabetes onset with DK/DKA among children and adolescents and followed up for two years, and further figures out the effective diagnostic metrics in effort to shed light on better classifying diabetes.

The results indicate that C-peptide, BMI, SBP and fatty liver at onset possess significant diagnostic value. These readily available clinical parameters provide a practical approach for classifying newly diagnosed diabetes onset with ketosis or ketoacidosis in pediatric populations.

**Supplementary Information:**

The online version contains supplementary material available at 10.1186/s12902-025-02077-x.

## Introduction

Diabetes is one of the most prevalent chronic diseases in childhood. According to the latest estimates, more than 1.2 million children and adolescents worldwide are living with diabetes, and the number of newly diagnosed cases each year is around 180,000 [1]. As guidelines recommended [2–4], the classification of diabetes in children and adolescents at diagnosis is typically based on their characteristics at presentation and genetic testing for the specific types. However, the increasing overlap in clinical features between type 1 (T1DM) and type 2 diabetes mellitus (T2DM) has made accurate diagnosis challenging [5].

Diabetic ketosis (DK) and ketoacidosis (DKA) occur commonly at the onset of diabetes and have traditionally been regarded as hallmark features of T1DM, particularly among children and adolescents, with reported DKA frequencies ranging from 15 ~ 70% [6, 7]. However, recent studies indicate that obese adolescents presenting with clinical features suggestive of T2DM may also develop DK/DKA, with prevalence rates of 5%–25% [8, 9]. Notably, some individuals with diabetes experience near-normoglycemic remission, which can persist for months to years. This clinical presentation of diabetes, also referred to as type 1B or atypical diabetes, is now widely accepted as ketosis-prone type 2 diabetes [10, 11]. It has also been well described among Black populations and is increasingly recognized in other high-risk groups, including Chinese, Japanese, and Hispanic individuals [12–14].

Accurate diabetes classification is essential for guiding treatment decisions, and thus, it is critical for clinicians to differentiate between the types of diabetes as early as possible. Although C-peptide, body mass index (BMI), and onset age had been proven to be effective diagnostic indicators for diabetes classification at newly diagnosis of diabetes onset with DK/DKA among adults, or among mixed cohorts of adults with the small proportion of youth included [15, 16]. However, it is important to note that the evidence in adults cannot be simply applied to children and adolescents since there are significant differences between adult-onset and pediatric diabetes, including pathophysiology, development, and treatment response. Furthermore, evidence regarding pediatric populations, particularly Asian children, remains limited. Current literature provides only partial insights into differentiating diabetes subtypes in children and adolescents presenting with new-onset DK/DKA, lacking specific data for Asian populations and well-defined, ethnicity-specific diagnostic criteria [17]. To address this gap, we retrospectively collected and analyzed clinical data from Chinese children and adolescents with new-onset DK/DKA over a two-year follow-up period. This study aimed to determine optimal diagnostic cut-off values for key discriminators, providing a quantitative framework for precise diabetes classification in this population.

## Methods

### Data source

**Fig. 1:**
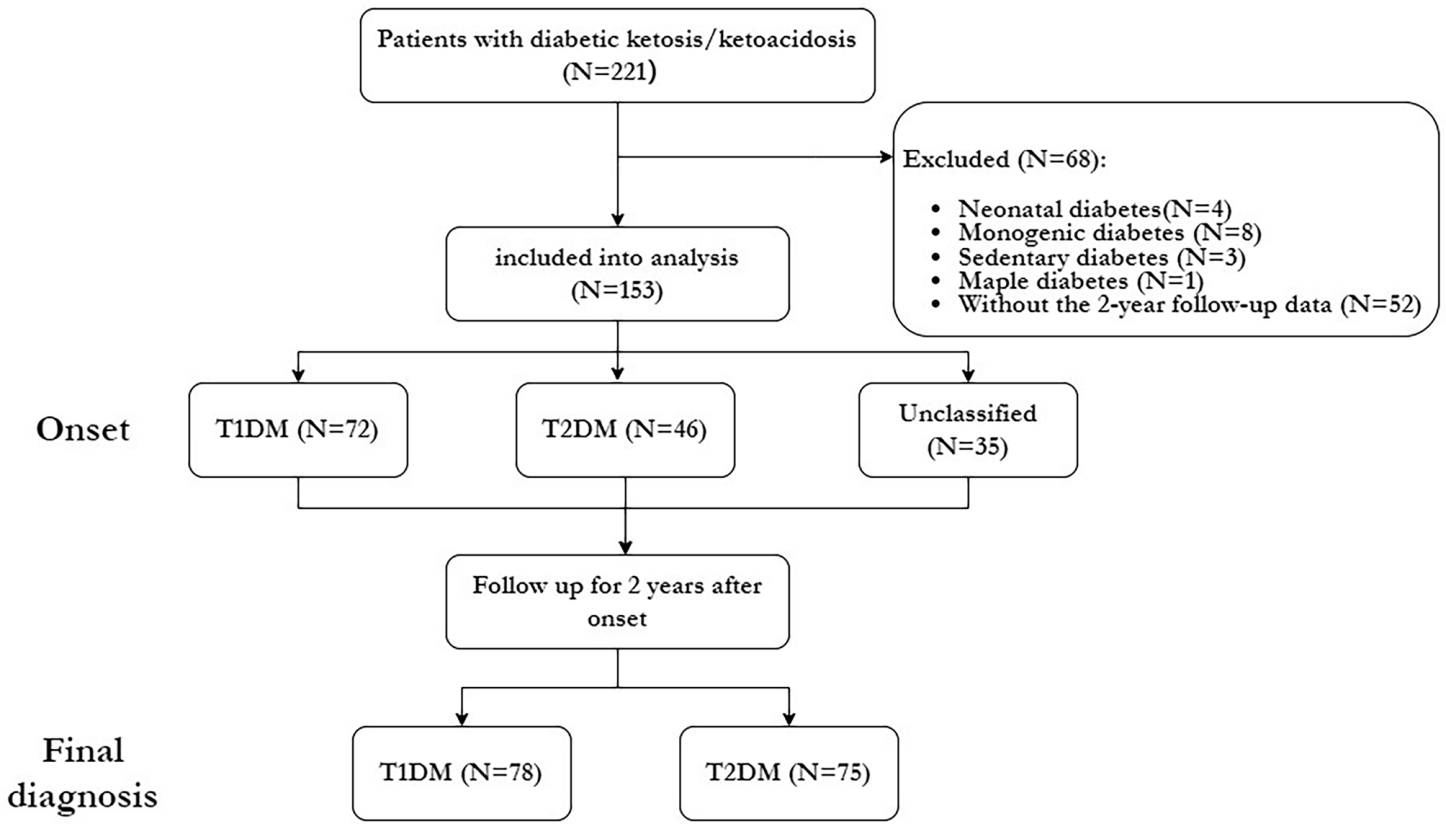
Flowchart of participants with diabetes selection. Abbreviations: T1DM, type 1 diabetes mellitus; T2DM, type 2 diabetes mellitus

This hospital-based, retrospective study collected data from newly diagnosed diabetes cases with ketosis or ketoacidosis at onset among children and adolescents at Henan Provincial People’s Hospital. Medical records of all episodes of DK/DKA from January 2017 to April 2020 were reviewed. A flowchart illustrating the participant selection process for the study is presented in Fig. 1. The study was conducted in accordance with the guidelines of the Declaration of Helsinki, and all procedures involving human subjects were approved by the Ethics Committee of Henan Provincial People’s Hospital.

## Participants

The inclusion criteria were as follows: (1) age at onset under 18 years; (2) diagnosed with diabetic ketoacidosis (DKA) based on the ICD code from the electronic health records (EHRs) or biochemical criteria for DKA as recommended by guidelines [2, 18]: a. hyperglycemia (blood sample glucose > 11 mmol/L [≈ 200 mg/dL]), serum blood bicarbonate < 18 mmol/L or pH < 7.30, ketonemia (blood β-hydroxybutyrate ≥ 3 mmol/L) or moderate or large ketonuria; those with hyperglycemia, ketonuria and ketonemia while without acidosis were also included due to the presence of DK; (3) availability of medical records both at onset and after two years of diabetes. Given that the primary objective of this study was the classification and diagnosis of diabetes in pediatric patients with DK/DKA onset, individuals with other types of diabetes, including drug-induced or pancreatitis-induced diabetes, as well as genetically induced diabetes without DKA manifestations (e.g., neonatal diabetes, MODY), were excluded from the study.

## Data collection

Data at the onset of diabetes were collected by endocrinologists from electronic health records (EHRs) using Excel. Information on demographic characteristics, physical examinations (BMI, systolic and diastolic blood pressure [SBP, DBP]), and medical histories related to diabetes (family history, precipitating factors for DK/DKA, presence of fatty liver, treatment, and any acute or chronic complications) were gathered. Biomedical data, including glucose levels at admission, glycated hemoglobin A1c (HbA1c), triglyceride (TG), total cholesterol (TC), serum uric acid (UA), serum creatinine (sCr), and high/low-density lipoprotein cholesterol (HDL-C, LDL-C) levels, were also collected. Additionally, the qualitative results for islet autoantibodies, including glutamic acid decarboxylase autoantibody (GADA), insulinoma-associated antigen-2 autoantibody (IA-2 A), insulin autoantibody (IAA), zinc transporter-8 autoantibody (ZnT8A), and islet-cell antibody (ICA), were recorded.

## Measurement

Emergency biochemical indicators, HbA1c, routine urinalysis, baseline C-peptide, and islet cell antibody tests are routinely performed upon admission for children with newly diagnosed diabetes presenting with DK/DKA. Corresponding corrective treatments for DKA or DK are administered in accordance with DKA management guidelines. Subsequently, intensive insulin therapy is initiated to regulate blood glucose levels and preserve islet cell function for 7 to 10 days (average 8.5 days) after correction of DK/DKA. Once blood glucose levels stabilize and meet the target, an oral glucose tolerance test (OGTT) with C-peptide release measurement is conducted.

Routine biochemical measurements of fasting blood samples were performed using an automatic analyzer (Architect C16000, Abbott, Chicago, USA). HbA1c was determined by high-performance liquid chromatography (Lifotronic H9, Lifotronic Instrument, Shenzhen, China; reference range: 4.0–6.1%). C-peptide was measured using enzyme-amplified chemiluminescence immunoassay (CLIA) technology (Immulite^®^ 2000 XPi Immunoassay System, Siemens Healthineers, Germany). GADA, IA-2 A, IAA, ZnT8A, and ICA were detected by radioligand binding assays, performed in duplicate. The cutoff indices for positivity were 10 units/mL (World Health Organization units) for GADA, IA-2 A, and ZnT8A, and 20 units/mL for IAA and ICA. The assay has been validated by the Islet Autoantibody Standardization Program 2012.

Abdominal ultrasonography was conducted by Technos Mindray equipment (Resona-8s, Shenzhen, China) with a convex probe operating at 1–6 MHz. Two independent experienced professional radiologists evaluated fatty liver ultrasonography findings included enhanced liver echogenicity, greater echogenicity in the liver compared to kidneys, deep attenuation and vascular blurring.

## Follow-up and classification

Information on treatment and diagnosis after two years was followed up via the medical records which collected in the outpatient and inpatient EHRs. Participants with diabetes were finally diagnosed with T1DM if they had a prior diagnosis of T1DM at the onset and continuously received basal-bolus insulin treatment during two years. Alternatively, participants with diabetes were also classified as having T1DM if they had a repeated C peptide below 0.6 nmol/L and received basal-bolus insulin treatment during the 2-year follow-up [19]. On the other hand, participants with diabetes were finally assigned to T2DM group if they had a previous diagnosis of diabetes and were managed solely with diet and exercise, or if they were prescribed oral hypoglycemic agents (OHAs) with or without basal insulin treatment, or they were treated with basal insulin only. Additionally, participants with diabetes who failed to comply with their insulin regimen for more than four weeks without experiencing recurrent DK/DKA at any point during their illness were also assigned to the T2DM group [20, 21].

## Statistical analysis

Statistical analyses were performed using SPSS 26.0 (Chicago, IL). Statistical significance was defined as a p-value < 0.05. Data are presented as means with standard deviations (SD) or medians with interquartile ranges (IQR) for normally distributed or skewed variables, respectively. Categorical variables are presented as counts (percentages). One-way analysis of variance (ANOVA) or the Mann–Whitney U-test was used for comparisons of quantitative variables between groups. The chi-squared test was performed to assess differences in proportions across groups. Receiver operating characteristic (ROC) curves and logistic regression analysis were used to evaluate the diagnostic performance of potential clinical characteristics, with sensitivities, specificities, and optimal cut-off points calculated for diabetes classification.

## Results

### Clinical presentations and distributions of included participants with diabetes

Table 1 summarizes the demographic and clinical characteristics of persons with DK/DKA at the onset of diabetes enrolled in this study. A total of 221 persons with medical records of DK/DKA when newly diagnosed as diabetes were included in the analysis, with 153 (69.23%) participants with diabetes meeting the inclusion criteria (Fig. 1). Among these 153 newly diagnosed diabetes onset with DK/DKA, the median age was 13.00 (11.00–15.00) years, with females accounting for 43.7% (67/153) of the sample. The median BMI was 21.30 (17.34–26.33) kg/m^2^, the median random glucose levels were 18.36 (15.00–22.85) mmol/L, and HbA1c levels were measured at 12.60 (11.10–14.40)%. In addition, specific descriptions of the precipitating factors for onset and the treatment at discharge are provided in the Appendix Supplementary (see appendix 1 - Table 1).

**Table 1:** Clinical characteristics of newly diagnosis of diabetes in children and adolescents with ketosis/ketoacidosis onset

	Total (*N* = 153)	T1DM (*n* = 72)	T2DM (*n* = 46)	Unclassified (*n* = 35)	F/χ2/H	*P*-value
**Gender (M/F) (n)**	86/67	32/40	34/12	20/15	9.92	0.007
**Age at the onset (yrs)**	13.00(11.00–15.00)	12.00(9.00–14.00)	14.50(12.75-17.00)	13.00(13.00–15.00)	19.804	< 0.001
**Family history (n)**	58(37.9)	17(23.6)	25(54.3)	16(45.7)	12.44	0.002
**BMI (kg/m** ^**2**^ **)**	21.30(17.34–26.33)	18.14(14.70-19.99)	27.02(24.81–29.23)	22.27(18.73–24.80)	77.487	< 0.001
**SBP (mmHg)**	112.00(103.00-123.00)	105.00(98.25–115.00)	124.00(120.00-131.50)	112.00(105.00-118.00)	52.787	< 0.001
**DBP (mmHg)**	72.00(65.00–79.00)	70.00(60.25-76.00)	75.50(68.50–80.00)	71.00(68.00–78.00)	6.504	0.039
**Hyperglycemia on admission**						
Random Glucose (mmol/L)	18.36 (15.00- 22.85)	19.88(16.63–25.37)	15.38(11.88–19.75)	18.36(15.91-24.00)	14.79	0.001
HbA1c (%)	12.60(11.10–14.40)	13.45(11.90-14.68)	11.70(10.08–13.43)	12.70(11.40–14.40)	12.645	0.002
**Lipids on admission**						
TC (mmol/L)	4.41 (3.82–5.09)	4.32(3.70–4.99)	4.58(4.03,5.29)	4.23(3.76–5.04)	3.51	0.173
TG (mmol/L)	1.35 (0.91–2.15)	1.06(0.80–1.61)	1.85(1.28–3.29)	1.34(0.81–2.42)	23.02	< 0.001
HDL-C(mmol/L)	1.05 (0.86–1.24)	1.10(0.88–1.37)	0.97(0.76–1.09)	1.05(0.90–1.28)	11.84	0.003
LDL-C(mmol/L)	2.62 ± 0.80	2.44 ± 0.71	2.92 ± 0.84	2.60 ± 0.81	5.52	0.005
Serum UA (umol/L)	282.5(217.5- 422.3)	239.0(179.5–307.0)	420.5(330.8-604.5)	260.0(226.0-369.0)	36.20	< 0.001
Serum Creatine (umol/L)	37.00 (31.00–49.00)	35.0(26.25–45.50)	42.00(34.75-53.00)	39.00(32.00–46.00)	0.61	0.544
**Beta-cell function**						
C peptide 0 h (nmol/L)	0.49(0.14–1.35)	0.15(0.10–0.32)	1.55(1.10–2.38)	0.68(0.36–1.27)	79.95	< 0.001
C peptide 0.5 h (nmol/L)	0.69(0.21–2.11)	0.21(0.10–0.33)	2.42(1.43–4.28)	1.01(0.52–1.97)	90.61	< 0.001
C peptide 1 h (nmol/L)	0.97(0.30–3.15)	0.30(0.10–0.62)	3.96(2.45–6.55)	1.42(0.79–3.01)	92.28	< 0.001
C peptide 2 h (nmol/L)	1.74(0.44–4.11)	0.44(0.15–0.95)	6.29(3.62–8.59)	1.84(1.12–3.42)	93.72	< 0.001
C peptide 3 h (nmol/L)	1.77(0.54–4.26)	0.54(0.17–1.08)	5.10(3.40–7.02)	2.12(1.22–3.66)	94.30	< 0.001
**Islet antibody ≥ 1 positive (%)**	31(20.3)	26(36.1)	2(4.3)	3(8.6)	21.37	< 0.001
GADA positive (%)	30(19.6)	25(34.7)	2(4.4)	3(8.6)	19.60	< 0.001
IAA positive (%)	1(0.7)	1(1.4)	0(0.0)	0(0.0)	1.12	0.572
ICA positive (%)	7(4.6)	6(8.3)	0(0.0)	1(2.9)	4.50	0.096
ZnT8 positive (%)	3(2.0)	2(2.8)	0(0.0)	1(2.9)	1.29	0.525
IA2Ab positive (%)	8(5.2)	7(9.7)	0(0.0)	1(2.9)	5.78	0.056
**Fatty Liver (%)**	52(34.0)	5(6.9)	38(82.6)	9(25.7)	73.01	< 0.001

Note: Data presented are mean ± SD, median (Q1–Q4), or n (%)

Abbreviations: T1DM: Type 1 diabetes mellitus; T2DM: Type 2 diabetes mellitus; BMI: Body mass index; SBP: Systolic blood pressure; DBP: Diastolic blood pressure; HbA1c: Glycated hemoglobin A1c; TC: Total cholesterol (reference range: 3.10-5.70 mmol/L); TG: Triglyceride (reference range: 0.34-1.92 mmol/L); HDL-C: High-density lipoprotein cholesterol (reference range: 0.78-2.00 mmol/L); LDL-C: Low-density lipoprotein cholesterol (reference range: 2.07-3.10 mmol/L); UA: Urine acid (reference range: 90-420 umol/L); GADA: Glutamate decarboxylase antibody; IAA: Insulin autoantibody; ICA: Islet-cell antibody; ZnT8A: Zinc transporter 8 autoantibody; IA2Ab: Islet antigen-2 (IA-2) autoantibody

### Clinical characteristics between groups after two years

After the two year follow-up, based on clinical manifestations, C-peptide levels, insulin treatment dependency, and other factors during follow-up, the final classification are as follows: there were 113 individuals with diabetes maintaining their initial diagnosis, with 67 (93.06%) diagnosed with T1DM and 46 (100.0%) diagnosed with T2DM. For those diagnosed with T1DM at onset, five persons were subsequently diagnosed with T2DM, among which two persons were treated with basal insulin in combination with OHAs, two persons were treated with OHAs alone, and the other one discontinued any anti-hyperglycemic treatment. For those with “un-classified” diabetes at onset (*N* = 35), 11 persons were ultimately diagnosed with T1DM, with the majority of them receiving basal-bolus insulin treatment and only one person receiving a combination of basal-bolus insulin and OHAs. The remaining 24 persons were finally diagnosed with T2DM, with 16 persons receiving OHAs, one person receiving GLP-1 treatment, three persons receiving a combination of basal insulin and OHAs, two persons maintaining unmedicated, and two persons maintaining basal insulin treatment only.

The between-group clinical characteristics after two years were shown in Table 2. Among those who were finally diagnosed with T1DM (*n* = 78) and T2DM (*n* = 75), it was observed that, compared to T2DM group, glycemia level was significantly higher in T1DM group, with a random glucose level of 19.60 (16.48–24.93) vs. 16.83 (13.80–22.00) mmol/L (*P* = 0.005) and a higher HbA1c value of 13.45(11.88–14.63) vs. 12.10(10.10–13.50) % (*P* = 0.001). Additionally, the positive rates of any islet antibody were also higher in the T1DM group (33.3% vs. 6.7%, *P* < 0.001), particularly the positive rate of GADA with 32.1% vs. 6.8% (*P* < 0.001). While for those finally diagnosed with T2DM, male persons predominated (66.7% vs. 46.2%, *P* = 0.011), and there was a significantly higher age at the onset (14.00[13.00–16.00] vs. 12.00[8.00–14.00] years, *P* < 0.001), BMI (18.07[14.52–19.89] vs. 25.97[22.68–28.34] kg/m^2^, *P* < 0.001), concomitant rate of fatty liver (64.0% vs. 5.1%, *P* < 0.001), percentage of family history of diabetes and blood pressure than T1DM group. In terms of β-cell function, T2DM had significantly higher C peptide levels in both fasting and within 3-h postprandial periods (*P* all < 0.05).

**Table 2:** Comparison of clinical characteristics between the two groups after follow-up

	T1DM	T2DM	t/χ^2^/Z	*P*-value
N (%)	78(51.0)	75(49.0)		
Gender (M/F)	36/42	50/25	6.536	0.011
Age at the onset (yrs)	12.00(8.00–14.00)	14.00(13.00–16.00)	-4.764	<0.001
Family history of diabetes (n/%)	16(20.5)	42(56.0)	20.457	<0.001
BMI (kg/m^2^)	18.07(14.52–19.89)	25.97(22.68–28.34)	-8.949	<0.001
SBP (mmHg)	105.00(98.00-115.00)	122.00(111.00-130.00)	-6.952	<0.001
DBP (mmHg)	70.00(61.75-76.00)	74.00(68.00–80.00)	-2.783	0.005
Random glucose (mmol/L)	19.60(16.48–24.93)	16.83(13.80–22.00)	-2.823	0.005
HbA1_C_ (%)	13.45(11.88–14.63)	12.10(10.10–13.50)	-3.202	0.001
TC (mmol/L)	4.25(3.68–4.85)	4.55(3.95–5.27)	-1.745	0.081
TG (mmol/L)	1.06(0.78–1.57)	1.78(1.17–2.85)	-4.654	<0.001
HDL-C (mmol/L)	1.07(0.88–1.37)	1.02(0.79–1.14)	-2.176	0.03
LDL-C (mmol/L)	2.45 ± 0.73	2.80 ± 0.82	-2.796	0.006
Serum UA (mmol/L)	237.00(180.5-303.25)	379.00(260.00-480.00)	-5.245	<0.001
Serum Creatine (mmol/L)	33.50(26.75-45.00)	42.00(34.00–52.00)	-3.591	<0.001
C peptide 0 h (nmol/L)	0.15(0.10–0.33)	1.35(0.73–2.20)	-9.411	<0.001
C peptide 0.5 h (nmol/L)	0.21(0.10–0.34)	2.11(1.27–4.04)	-9.975	<0.001
C peptide 1 h (nmol/L)	0.32(0.10–0.64)	3.13(1.62–5.20)	-9.864	<0.001
C peptide 2 h (nmol/L)	0.50(0.16–1.04)	3.95(2.43–7.33)	-9.574	<0.001
C peptide 3 h (nmol/L)	0.54(0.17–1.16)	4.18(2.59–6.36)	-9.714	<0.001
Positive rate of any islet antibody (%)	26(33.3)	5(6.7)	16.829	<0.001
GADA positive (%)	25(32.1)	5(6.8)	15.337	<0.001
IAA positive (%)	1(1.3)	0(0.0)	0.955	0.328
ICA positive (%)	7(9.0)	0(0.0)	6.962	0.008
ZnT8 positive (%)	3(3.8)	0(0.0)	2.903	0.088
IA2Ab positive (%)	8(10.3)	0(0.0)	8.011	0.005
Fatty Liver (%)	4(5.1)	48(64.0)	59.066	<0.001
Precipitating factors (%)	58(74.4)	59(78.7)	0.394	0.530

Note: Data presented are mean ± SD, median (Q1–Q4), or n (%);

Abbreviations: T1DM: Type 1 diabetes mellitus; T2DM: Type 2 diabetes mellitus; BMI: Body mass index; HbA1c: Glycated hemoglobin A1c; TC: Total cholesterol (reference range: 3.10–5.70 mmol/L); TG: Triglyceride (reference range: 0.34–1.92 mmol/L); HDL-C: High-density lipoprotein cholesterol (reference range: 0.78-2.00 mmol/L); LDL-C: Low-density lipoprotein cholesterol (reference range: 2.07–3.10 mmol/L); UA: Urine acid (reference range: 90–420 umol/L); GADA: Glutamate decarboxylase antibody; IAA: Insulin autoantibody; ICA: Islet-cell antibody; ZnT8A: Zinc transporter 8 autoantibody; IA2Ab: Islet antigen-2 (IA-2) autoantibody

### ROC curves and logistic regression analysis for classifying diabetes

**Fig. 2:**
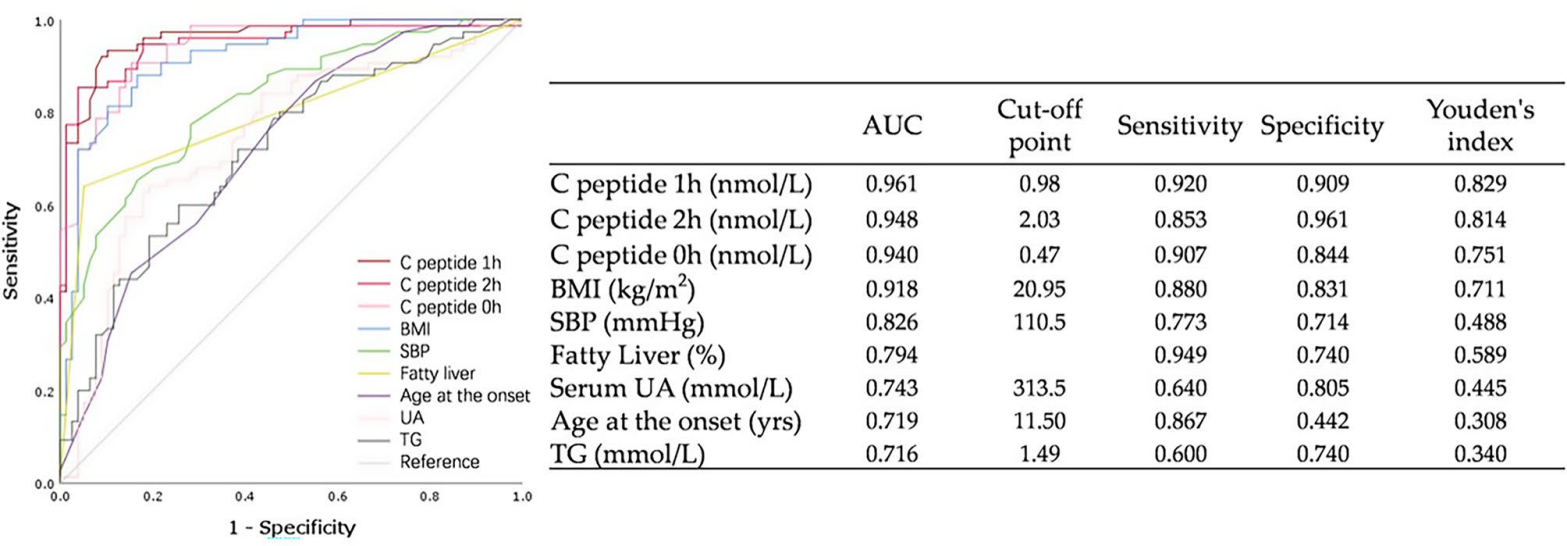
ROC analysis for classifying diabetes among all participants with DK/DKA. Abbreviations: T1DM, type 1 diabetes mellitus; T2DM, type 2 diabetes mellitus. BMI: Body mass index; TG༚Triglyceride (refer-ence range: 0.34–1.92 mmol/L); UA: urine acid (reference range: 90–420 umol/L)

The performance of each metric in classifying diabetes, as indicated by the estimated AUC and its 95% confidence interval (CI), is presented in Fig. 2. β-cell function, as measured by the C-peptide level within 3 h, exhibited the highest AUC values for diabetes classification, ranging from 0.940 (95% CI: 0.903–0.976) to 0.961 (95% CI: 0.932–0.989) (*P* < 0.05 for all). BMI, with an optimal cut-off point of 20.95 kg/m², demonstrated significant prognostic value, with an AUC of 0.918 (95% CI: 0.874–0.962), and sensitivity and specificity of 88.0% and 83.1%, respectively. Both systolic blood pressure (SBP) and the presence of fatty liver showed significant prognostic value, with AUCs of 0.826 (95% CI: 0.761–0.890) and 0.794 (95% CI: 0.720–0.869), respectively. The AUCs for serum uric acid (UA), age at onset, and triglycerides (TG) in diabetes classification were significantly moderate, with values gradually decreasing from 0.743 (95% CI: 0.663–0.824) for UA, 0.719 (95% CI: 0.638–0.799) for age, to 0.716 (95% CI: 0.635–0.797) for TG (*P* < 0.05). In contrast, other metrics, such as gender, family history of diabetes, presence of islet autoantibodies, diastolic blood pressure (DBP), total cholesterol (TC), HDL-C/LDL-C ratios, and creatinine, exhibited poor performance, with AUCs all less than 0.7, and no prognostic ability. Logistic regression analysis further confirmed that BMI (*P* = 0.003), SBP (*P* = 0.042), and C-peptide levels at 0 h (*P* = 0.046) and 0.5 h (*P* = 0.001) were significantly effective in classifying diabetes.

## Discussion

This study is aimed to investigate clinical indicators that can effectively differentiate diabetes types in pediatric newly diagnosed with DK/DKA, and to quantify diagnostic cut-off values for key clinical parameters to accurately classify diabetes subtypes in this unique Chinese pediatric and adolescent cohort presenting with new-onset DK/DKA. We demonstrated that C peptide, BMI, SBP, and presence of fatty liver are robust discriminators. Most importantly, we established population-specific optimal cut-off points for each indicator. These quantifiable thresholds provide an objective tool to aid clinical decision-making in cases with overlapping features.

The absolute or relative deficiency of insulin, along with the impairment of β-cell function, is recognized as a cornerstone of diabetes pathophysiology. Therefore, assessment of insulin secretory capacity is instrumental in classifying diabetes types and guiding treatment strategies. C-peptide, secreted in equimolar amounts with insulin, differs from insulin in that it is not extracted by the liver; hence, its measurement is unaffected by insulin therapy. It has now emerged as a valuable clinical biomarker for assessing β-cell function to clarify uncertain diagnoses, particularly in populations already receiving insulin treatment [22].

In this study, similar to previous studies conducted among both adults and pediatric populations [12, 14, 23], the levels of C-peptide remained the most effective value for distinguishing between T1DM and T2DM during the entire duration of the 3-hour tests. Notably, our study established higher C-peptide cut-off values than previous reports - specifically, 0.47 nmol/L (fasting) and 2.03 nmol/L (2-hour postprandial), compared to adult studies reporting approximately 0.2 nmol/L (fasting) and 0.5 nmol/L (2-hour postprandial) [15]. Published evidence confirms that C-peptide levels at diagnosis exhibit age-dependent increases within each BMI stratum [24–27], independent of adiposity effects. These findings suggest that the observed discrepancy likely reflects age-related physiological variations, given that our cohort represents the youngest population with diabetes studied to date. Importantly, C-peptide levels may initially rise during the diagnosis of pediatric T1DM, leading to a remission period, but subsequently decline rapidly in the first years after diagnosis. Therefore, implementing appropriately elevated C-peptide thresholds could improve diagnostic precision in classification. It is crucial to increase focus on pediatric patients who retain moderate C-peptide levels.

Overweight has been demonstrated to accelerate progression to T1DM in children and adolescents, in addition to its well-known association with T2DM. In this study, consistent with prior research on adults with ketosis-prone diabetes [28], BMI was confirmed as a significant classifier between T1DM and T2DM in youth presenting with DK/DKA at onset, ranking second only to C-peptide in ROC analysis. Furthermore, unlike most previous studies [13, 14], we additionally incorporated fatty liver rate as a measure of visceral adiposity rather than relying solely on BMI (which primarily reflects subcutaneous fat). Our findings indicate that fatty liver indeed holds substantial discriminative value for diabetes classification, particularly in obesity-related diabetes types. These results underscore the critical need for precise diabetes type differentiation in the context of childhood obesity, given its growing contribution to the rising prevalence of diabetes in pediatric populations.

The autoimmune antibodies, a hallmark feature of T1DM, have been reported positive in several youths with clinically diagnosed T2DM [29]. Since the recognition of ketosis-prone diabetes, antibody positivity has served as a key diagnostic criterion for diabetes classification. However, in this study, while we observed a statistically significant difference in islet antibody positivity rates between T1DM and T2DM cases, the limited sample size likely reduced its discriminative power in ROC analysis. Furthermore, as presented by previous studies, the islet of antibodies can also be negative in up to 20% of otherwise classical T1DM, particularly in children. The reported lower sensitivity or false negative rate may also explain our observations.

A strength of the present study is the inclusion of a relatively large participants in both the confirmation and prognosis cohort, which had significantly reduced the selection bias. Additionally, we conducted post-hoc analyses comparing clinical characteristics at disease onset across cohorts. Furthermore, we not only compared clinical characteristics at onset but also established specific cut-off values for key discriminators, enhancing the clinical applicability of our findings. The metrics used in this study are routinely available in clinical practice, further increasing the practical utility of the results. However, the study has several limitations. Its retrospective design meant that important data, such as detailed islet antibody profiles, were often incomplete or missing after the two-year follow-up, which may introduce bias. Additionally, a significant number of pH and bicarbonate values were missing in our data collection, partly due to the inherent challenges of performing arterial blood gas analysis in pediatric patients. Furthermore, the limited availability of genetic testing for monogenic diabetes in China may lead to diagnostic misclassification, which could influence the interpretation of our findings. Another limitation of this study is that the study is based on data collected from a single tertiary medical institution, which predominantly treats patients with more severe or atypical cases. Consequently, there is a potential for selection bias, which may limit the generalizability of our findings to the broader population of individuals with diabetes, particularly those with milder forms or more typical clinical presentations. The institution is likely to admit patients with more severe complications, and this selection bias may skew the results, especially within the pediatric and adolescent diabetes cohort. Additionally, as a single-center study, the patient population is inherently more limited and may not fully capture the clinical spectrum of diabetes across different regions and healthcare settings. Therefore, future studies should consider multi-center designs that include a more diverse patient population to enhance the external validity and generalizability of the findings.

## Conclusions

In individuals with ambiguous clinical features, where misclassification of diabetes type occurs at initial presentation, the time between presentation and correct classification presents significant potential risks. Therefore, this study estimates the most sensitive and specific clinical and biochemical criteria for the diagnostic classification of newly diagnosed diabetes with ketosis or ketoacidosis at onset in pediatric patients. Caution is required when interpreting C-peptide levels, BMI, systolic blood pressure (SBP), and fatty liver at onset, as these metrics have effective diagnostic value.

## Supplementary Information

Below is the link to the electronic supplementary material.

Supplementary Material 1

## Data Availability

The authors confirm that the majority of the data supporting the findings of this study are available within the article. Raw data are available from the authors upon reasonable request. The data are not publicly available due to privacy restrictions.
