# Supplementary material for: Differentiating diabetes type in children and adolescents with ketosis or ketoacidosis at onset: a retrospective analysis of clinical and biochemical markers
Source: BMC Endocr Disord. 2025 Nov 12;25:258. doi: 10.1186/s12902-025-02077-x (PMC12613522; doi:10.1186/s12902-025-02077-x)
Supplement: Supplementary file 1 — Supplementary Material 1 [file 12902_2025_2077_MOESM1_ESM.docx]

**Supplementary appendix 1.**

**Table 1.** Clinical characteristics of newly diagnosis of diabetes in children and adolescents with ketosis/ketoacidosis onset.

|  | Total (N=153) | T1DM (n=72) | T2DM (n=46) | Unclassified (n=35) | F/χ2/H | P-value |
| --- | --- | --- | --- | --- | --- | --- |
| **Precipitating factors (%)** | 117(76.5) | 55(76.4) | 37(80.4) | 25(71.4) | 0.90 | 0.639 |
| **Treatment at discharge** | | | | | | |
| Insulin (%) | 99(64.7) | 68 (94.4) | 9 (19.6) | 22 (62.9) | 91.69 | <0.001 |
| Insulin +OHA (%) | 17(11.1) | 2 (2.8) | 10 (21.7) | 5 (14.3) |  |  |
| OHA (%) | 21(13.7) | 2 (2.8) | 16 (34.8) | 3 (8.6) |  |  |
| GLP-1(%) | 10(6.5) | 0 (0.0) | 10 (21.7) | 0 (0.0) |  |  |
| Drugs withdrawal (%) | 6(3.9) | 0 (0.0) | 1 (2.2) | 5 (14.3) |  |  |

Note: Data presented are mean ± SD, median (Q1–Q4), or n (%);

Abbreviations: T1DM: Type 1 diabetes mellitus; T2DM: Type 2 diabetes mellitus; OHA: Oral-hypoglycemic agents; GLP-1: Glucagon-like peptide-1.
